# Clinicopathologic Spectrum and Oxford MEST-C Scores in Adult IgA Nephropathy in Middle Eastern Cohorts: A PRISMA-Guided Literature Review With Crude Pooled Lesion Frequencies

**DOI:** 10.7759/cureus.100555

**Published:** 2026-01-01

**Authors:** Fahad S Alrashidi, Maha z Alrasheedi

**Affiliations:** 1 Internal Medicine/Nephrology, Ad Diriyah Hospital, Riyadh Third Health Cluster, Riyadh, SAU; 2 Family Medicine, Riyadh Second Health Cluster, Riyadh, SAU

**Keywords:** clinicopathologic spectrum, iga nephropathy, middle east, oxford mest-c, renal outcomes

## Abstract

IgA nephropathy (IgAN) is the most common primary glomerulonephritis worldwide, but Middle Eastern adults are under-represented in cohorts reporting Oxford MEST-C (mesangial, endocapillary, segmental, tubular atrophy/interstitial fibrosis, and crescent) lesions, limiting region-specific histologic risk profiling. We performed a Preferred Reporting Items for Systematic Reviews and Meta-Analyses (PRISMA)-guided systematic review of observational adult (≥18 years) primary IgAN cohorts from Middle Eastern countries reporting Oxford MEST or MEST-C. MEDLINE (PubMed), Embase, and Scopus were searched from January 1, 2009, to November 24, 2025, with no language restrictions; citation searching (backward reference screening and forward citation tracking) was also performed. Two reviewers independently screened studies, extracted data, and assessed risk of bias using the Newcastle-Ottawa Scale (NOS). For each MEST-C component, we calculated descriptive pooled proportions by summing numerators and denominators across cohorts with available extractable data; renal outcomes were synthesized narratively. Four retrospective cohorts (total n=528) from Iran, Turkey, and Saudi Arabia met eligibility criteria. Three cohorts contributed extractable lesion counts for M1, S1, and T1-2 (E1 data were available in two cohorts). Descriptive pooled frequencies were: mesangial hypercellularity (M1) 80.0%, endocapillary hypercellularity (E1) 35.7%, segmental glomerulosclerosis (S1) 58.5%, and tubulointerstitial scarring (T1-2) 56.4%. Crescent reporting was limited; where extractable, C≥1 was 28.9% in the Turkish cohort, and a sensitivity approximation incorporating a study reporting overall crescent frequency yielded ~27.6%. Where reported, T and crescent lesions were associated with worse renal outcomes. Formal heterogeneity testing and quantitative outcome meta-analysis were not performed due to a few clinically diverse cohorts. Risk of bias was low-to-moderate, mainly due to retrospective design, convenience sampling, and incomplete outcome reporting. Available Middle Eastern cohorts show frequent M1 and S1, with substantial T1-2 scarring and crescents in a sizeable minority of biopsied patients. These findings likely reflect advanced histologic injury at biopsy in tertiary-care cohorts and/or variation in case mix and biopsy/referral practices, underscoring the need for prospective regional registries with standardized pathology reporting and outcome definitions.

## Introduction and background

IgA nephropathy (IgAN) is the leading cause of primary glomerulonephritis worldwide and a major contributor to chronic kidney disease (CKD) and end-stage kidney disease (ESKD) among young and middle-aged adults. Clinically, it manifests with microscopic or macroscopic hematuria, variable proteinuria, and progressive kidney function decline in a substantial proportion of patients. Histologically, IgAN is defined by dominant or co-dominant mesangial deposition of immunoglobulin A (IgA) on immunofluorescence, accompanied by mesangial and, variably, endocapillary hypercellularity, segmental sclerosis, chronic tubulointerstitial scarring, and crescents [1-4].

In 2009, the Oxford classification was introduced as a standardized grading system for IgAN, incorporating four key histologic lesions: mesangial hypercellularity (M), endocapillary hypercellularity (E), segmental sclerosis (S), and tubular atrophy/interstitial fibrosis (T). The classification was subsequently updated to include crescents (C), forming the mesangial, endocapillary, segmental, tubular atrophy/interstitial fibrosis, and crescents (MEST-C) score. Multiple studies from Europe, East Asia, and North America have confirmed that individual MEST-C components - especially tubular atrophy/interstitial fibrosis (T) lesions and crescents (C) - carry independent prognostic information for kidney outcomes and are central to risk stratification [5-11].

Patients from Middle Eastern countries are under-represented in the cohorts that informed the original Oxford classification and in subsequent validation and prediction model studies. Biopsy practices, referral patterns, genetic background, and comorbidity profiles may differ in the Middle East compared with Western and East Asian populations, potentially influencing both clinicopathologic presentation and progression of IgAN. Single-center and multicenter reports from the region suggest that many patients present with advanced CKD and heavy proteinuria, but these data are scattered and have not been synthesized systematically [7-11].

To address this gap, we conducted a Preferred Reporting Items for Systematic Reviews and Meta-Analyses (PRISMA)-guided systematic literature review using crude pooled lesion frequencies to (1) describe the clinicopathologic profile of adult patients with primary IgAN in Middle Eastern cohorts and (2) summarize the crude pooled frequencies of individual Oxford MEST-C components among biopsy-proven patients. This review was explicitly not designed to estimate country-level or population-level incidence or prevalence of IgAN, nor to perform formal meta-analytic modeling of treatment effects or outcomes.

## Review

Methods

Study Design and Registration

We conducted a PRISMA-guided systematic literature review of observational cohort and biopsy studies. The a priori aim was to provide a descriptive regional synthesis of clinicopathologic patterns rather than a population-based prevalence study. Because the included cohorts were retrospective, center-based convenience samples and few in number, quantitative synthesis was restricted to crude pooled frequencies of histologic lesions among biopsied patients; we did not attempt formal meta-analytic modeling of incidence, prevalence, treatment effects, or outcomes. The protocol was registered in the International Prospective Register of Systematic Reviews (PROSPERO; CRD420251184273).

Eligibility Criteria

We included studies that fulfilled all of the following criteria: (1) adult patients (≥18 years) with biopsy-proven primary IgAN; (2) nephrology centers, pathology services, or kidney biopsy registries from Middle Eastern countries (e.g., Saudi Arabia, Kuwait, Qatar, United Arab Emirates, Oman, Bahrain, Jordan, Lebanon, Syria, Iraq, Yemen, Egypt, Iran, Turkey); (3) kidney biopsies evaluated using the Oxford classification (mesangial, endocapillary, segmental, tubular atrophy/interstitial fibrosis, and crescents (MEST-C) or the earlier MEST score), with extractable data for at least one MEST-C component (M, E, S, T, or C); (4) observational cohort (retrospective or prospective) or cross-sectional biopsy series with ≥10 adult patients; and (5) reporting at least clinicopathologic characteristics and/or histologic lesion frequencies. We excluded pediatric-only cohorts; very small case series; studies of secondary IgA deposition without separable primary IgAN data; mixed glomerular disease cohorts without extractable IgAN-specific outcomes; animal/experimental studies; and conference abstracts with insufficient data. The eligibility criteria are summarized in Table 1.

**Table 1:** Inclusion and exclusion criteria for the study selection IgAN = immunoglobulin A nephropathy; MEST-C = mesangial, endocapillary, segmental, tubular atrophy/interstitial fibrosis, and crescents

Inclusion criteria	Exclusion criteria
Adult patients (≥18 years) with biopsy-proven primary IgA nephropathy (IgAN).	Pediatric-only cohorts (<18 years).
Nephrology centers, pathology services, or kidney biopsy registries from Middle Eastern countries (e.g., Saudi Arabia, Kuwait, Qatar, United Arab Emirates, Oman, Bahrain, Jordan, Lebanon, Syria, Iraq, Yemen, Egypt, Iran, Turkey).	Cohorts from non–Middle Eastern countries.
Kidney biopsies evaluated using the Oxford classification (MEST or MEST-C), with extractable data for at least one MEST-C component (M, E, S, T, or C).	Studies of secondary IgA deposition without separately reported primary IgAN data.
Observational cohort (retrospective or prospective) or cross-sectional biopsy series with ≥10 adult patients.	Very small case series with <10 adult patients.
Reporting at least clinicopathologic characteristics and/or histologic lesion frequencies (e.g., M1, E1, S1, T1–2, C1–2).	Mixed glomerular disease cohorts where IgAN-specific data could not be isolated, animal or experimental models, and conference abstracts without adequate data.

Information Sources and Search Strategy

We searched MEDLINE (PubMed), Embase, and Scopus from January 1, 2009, to November 24, 2025. Search concepts included (1) IgA nephropathy (“Glomerulonephritis, IgA” OR “IgA nephropathy” OR “Berger disease”), (2) Oxford classification terms (Oxford OR MEST OR MEST-C), and (3) Middle Eastern country names; searches were limited to human studies, with no language restrictions. We additionally screened reference lists of eligible studies and relevant reviews (backward citation searching) and performed forward citation tracking in Google Scholar or Web of Science to identify newer reports. Full database-specific search strategies are provided in the Appendix/Supplementary Methods. [1,2,12].

Study Selection

**Figure 1:**
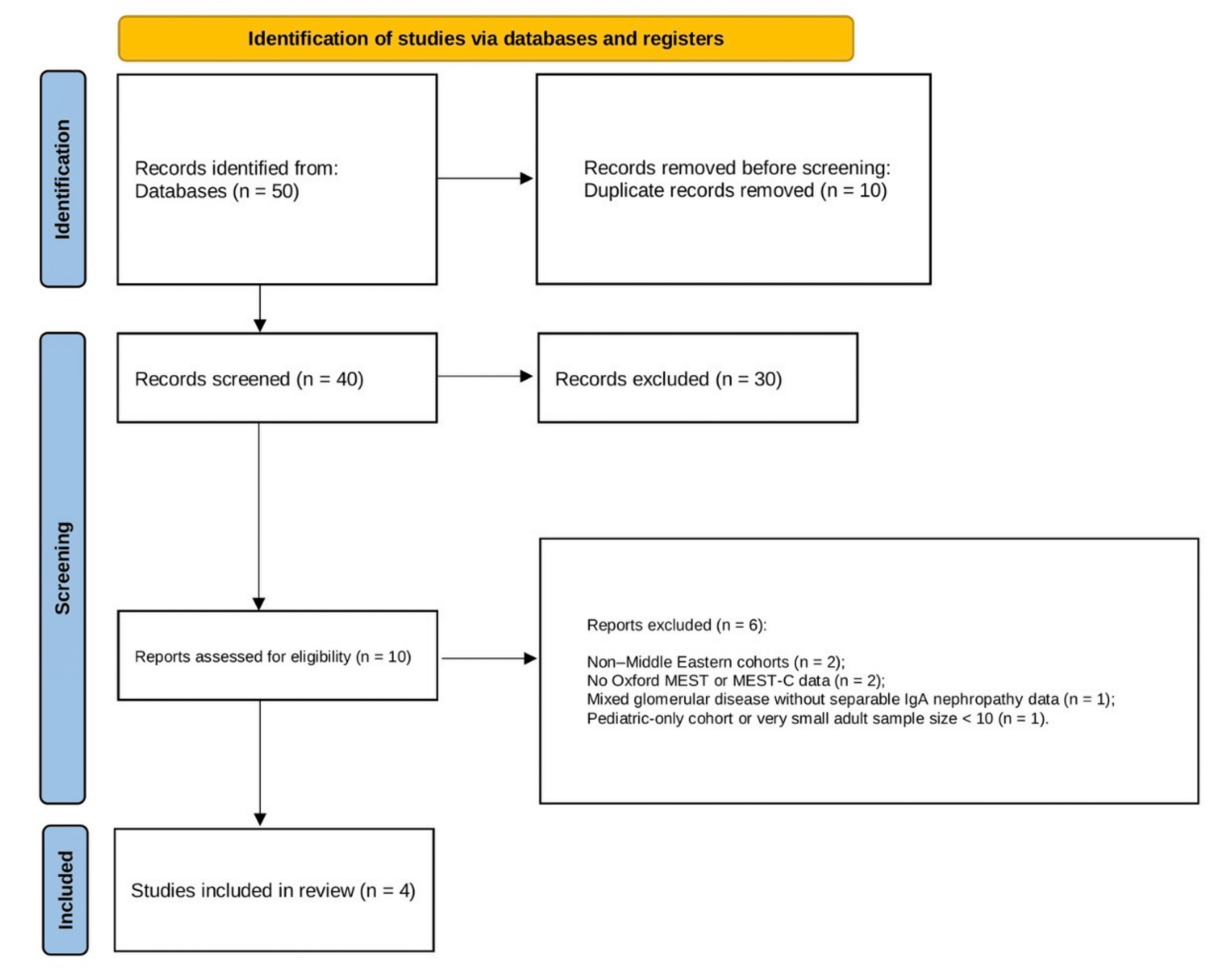
PRISMA 2020 flow diagram for the study selection Flow diagram showing identification, screening, eligibility assessment, and inclusion of studies in the systematic review. A total of 50 records were identified, 10 duplicates were removed, 40 records were screened, 10 full-text reports were assessed for eligibility, six were excluded with reasons, and four studies were included in the final review. PRISMA: Preferred Reporting Items for Systematic Reviews and Meta-Analyses

All records retrieved from database searches were imported into a reference manager, and duplicates were removed. Two reviewers independently screened titles and abstracts against the eligibility criteria, classifying each record as “include,” “exclude,” or “uncertain.” Full-text articles were obtained for all records not clearly excluded. The same two reviewers then independently assessed full texts for eligibility, with disagreements resolved by discussion and consensus. Reasons for exclusion at the full-text stage were recorded. The study selection process is summarized in a PRISMA 2020 flow diagram (Figure 1) [12].

Data Extraction

A standardized data extraction form in Microsoft Excel (Microsoft® Corp., Redmond, WA) was used. Two reviewers independently extracted study characteristics (country, center type, study design, biopsy period, sample size); baseline demographics (age, sex); baseline clinical data (estimated glomerular filtration rate (eGFR), serum creatinine, degree of proteinuria, prevalence of hypertension and diabetes, and follow-up duration where applicable); histologic findings according to the Oxford classification (numbers of patients with mesangial hypercellularity (M1), endocapillary hypercellularity (E1), segmental sclerosis (S1), tubular atrophy/interstitial fibrosis grades 0-2 (T0, T1, T2), and crescents (C0, C1, C2)); and renal outcomes where available. When only percentages were provided, counts were derived by multiplying by the sample size and rounding to the nearest integer. When essential data were missing or unclear, we planned to contact the study authors for clarification.

Risk of Bias Assessment

Risk of bias for observational cohort and cross-sectional studies was assessed independently by two reviewers using the Newcastle-Ottawa Scale (NOS), evaluating the domains of selection, comparability, and outcome. Discrepancies were resolved by consensus. An overall risk-of-bias judgment (low, moderate, or high) was assigned to each study. We also considered risk of bias due to missing results or selective outcome reporting, particularly for Oxford mesangial, endocapillary, segmental, tubular atrophy/interstitial fibrosis, and crescents (MEST-C) components and renal endpoints [6,13].

Data Synthesis and Statistical Analysis

Given the descriptive aim and few heterogeneous cohorts, we report binomial 95% confidence intervals (Wilson) for the descriptive pooled proportions to indicate precision; however, we did not perform random-effects meta-analysis, formal statistical heterogeneity metrics (e.g., I²/τ²), or meta-regression, and outcomes were synthesized narratively [5-7].

Results

Study Selection

The search identified a few adult IgAN cohorts from Middle Eastern countries reporting Oxford MEST/MEST-C data. Overall, 45 records were retrieved from database searching and five additional records from citation searching (reference screening and forward citation tracking). After de-duplication, 40 unique records were screened; 10 full texts were assessed, and six were excluded (non-Middle Eastern cohorts; no Oxford MEST/MEST-C data; mixed glomerular disease without extractable IgAN-specific data; pediatric-only populations; or very small adult samples). Four adult cohorts met the inclusion criteria (Saudi Arabia, two from Iran, and a multicenter high-risk cohort from Turkey). Three studies provided extractable counts for individual MEST-C components, while one Iranian validation study focusing on interobserver agreement was included qualitatively. Study selection is summarized in Figure 1 (PRISMA 2020), and baseline cohort characteristics are summarized in Table 2.

**Table 2:** Study characteristics of included Middle Eastern adult IgA nephropathy cohorts IgAN = immunoglobulin A nephropathy; MEST = mesangial, endocapillary, segmental, tubular atrophy/interstitial fibrosis; TSN-GOLD = Turkish Society of Nephrology Glomerular Diseases Working Group; IST = immunosuppressive therapy; NR = not reported

Study ID (country)	Setting/center type	Study period*	Design	Sample size (n)	Notes
Nasri 2012 (Iran) [7]	Single nephrology/pathology center	~2000–2010 (NR)	Retrospective observational biopsy cohort	102	Adult primary IgA nephropathy (IgAN) with Oxford MEST scoring
Oruç 2024 (Turkey; TSN-GOLD) [9]	Multicenter nephrology network (Turkish Society of Nephrology Glomerular Diseases Working Group)	~2010–2020 (NR)	Retrospective cohort of high-risk IgAN treated with immunosuppressive therapy (IST)	408	Adults with biopsy-proven primary IgAN receiving immunosuppressive therapy
Bokhary 2021 (Saudi Arabia) [10]	Single tertiary care center	~2010–2020 (NR)	Retrospective clinicopathologic series	18	Adult biopsy-proven primary IgAN evaluated using the Oxford MEST-C classification

Study and Patient Characteristics

The four included studies comprised 528 adult patients with biopsy-proven primary immunoglobulin A nephropathy (IgAN) from Saudi Arabia, Iran, and Turkey. Sample sizes ranged from 18 patients in the Saudi clinicopathologic series to 408 patients in the Turkish multicenter cohort. Most cohorts were retrospective single-center or multicenter biopsy-based observational studies. The Turkish cohort enrolled adults with biopsy-proven primary IgAN who received immunosuppressive therapy in participating centers, whereas the Iranian and Saudi cohorts primarily described patients at the time of kidney biopsy with limited longitudinal follow-up. One Iranian study specifically evaluated the reproducibility of the Oxford classification across renal pathologists rather than focusing on lesion distributions [11,14-17].

Across studies, IgAN in this region predominantly affected young and middle-aged adults, and there was a consistent male predominance. Study-level characteristics and baseline clinical features at biopsy are summarized in Tables 2-3, respectively.

**Table 3:** Baseline clinical characteristics of the included Middle Eastern adult IgA nephropathy cohorts † At time of kidney biopsy or study enrollment, as reported. IgAN = immunoglobulin A nephropathy; eGFR = estimated glomerular filtration rate; IST = immunosuppressive therapy; TSN-GOLD = Turkish Society of Nephrology Glomerular Diseases Working Group; NR = not reported; SD = standard deviation

Study ID (country)	Sample size (n)	Mean age, years (±SD)	Male, %	Baseline kidney function†	Proteinuria (g/day)†	Follow-up duration	Comments
Nasri 2012 (Iran) [7]	102	37.7 ± 13.6	71.6	NR	NR	Minimal (cross-sectional at biopsy)	Iranian adult biopsy cohort
Oruç 2024 (Turkey; TSN-GOLD) [9]	408	38.4 ± 12.5	65.4	Median eGFR ≈ 68 mL/min/1.73 m²	Median proteinuria ≈ 2.8 g/day	~2.5 years (median)	High-risk IST-treated IgAN cohort
Bokhary 2021 (Saudi Arabia) [10]	18	24.0 ± 14.2	66.7	NR	NR	Cross-sectional (no long-term follow-up)	Saudi single-center clinicopathologic series

Baseline Clinical Features

Quantitative synthesis of baseline laboratory parameters was limited by heterogeneous reporting. In general, the mean age at biopsy was in the mid-30s in the Iranian cohorts and younger (24 years) in the Saudi series, and between 65% and 72% of patients were male across studies. In the large Turkish cohort, median serum creatinine was approximately 1.2 mg/dL and median eGFR about 68 mL/min/1.73 m², with high levels of proteinuria (median ≈ 2.8 g/day), indicating a considerable burden of glomerular injury at inclusion. Data on hypertension and diabetes mellitus were variably reported but suggested a sizeable burden of comorbidity, particularly hypertension. More detailed pooling of eGFR, proteinuria, and comorbidities would require harmonization of additional cohorts and extraction of data from tables not fully available in text form [9,18].

Histopathologic Findings (Oxford MEST-C)

Three cohorts (Iranian, Turkish, and Saudi; total n = 528) provided usable counts for at least some components of the Oxford mesangial, endocapillary, segmental, tubular atrophy/interstitial fibrosis, and crescents (MEST-C) classification. The prevalence of mesangial hypercellularity (M1) ranged from 65.7% in the Iranian cohort (67/102) to 84.1% in the Turkish cohort (343/408), with the Saudi series reporting M1 in 66.7% (12/18) of patients. Crude pooling across the three cohorts yielded a prevalence of 80.0% (422/528) for M1, indicating that mesangial hypercellularity is nearly universal in available Middle Eastern IgAN cohorts [7,9,10].

Endocapillary hypercellularity (E1) was reported numerically in the Iranian and Turkish cohorts. E1 was present in 32.4% of Iranian patients (33/102) and 36.5% of Turkish patients (149/408), yielding a pooled prevalence of 35.7% (182/510) across these two cohorts. The Saudi cohort presented endocapillary lesions only graphically, without exact counts, and therefore could not be included in pooled estimates for E1 [7,9,10].

Segmental sclerosis (S1) affected 66.7% (68/102) of patients in the Iranian cohort, 57.6% (235/408) in the Turkish cohort, and 33.3% (6/18) in the Saudi cohort. The crude pooled prevalence of S1 was 58.5% (309/528), indicating that more than half of adult IgAN patients in these Middle Eastern cohorts had segmental sclerosis at the time of biopsy [7,9,10].

Tubular atrophy/interstitial fibrosis grades 0-2 (T0, T1, T2) were reported in detail in the Iranian and Turkish cohorts, and T1/T2 combined in the Saudi cohort. In Iran, T0, T1, and T2 were observed in 51%, 30%, and 19% of patients, respectively; in Turkey, the corresponding proportions were 40.4%, 52.0%, and 7.6%. In the Saudi series, T1-2 combined were present in five of 18 patients (27.8%). When T1 and T2 were combined across all cohorts, the crude pooled prevalence of T1-T2 was 56.4% (298/528), underscoring the high burden of chronic tubulointerstitial scarring [7,9,10].

Data on crescents were more variable. In the Iranian cohort, any extracapillary proliferation (C≥1) was present in 22.5% (23/102) of patients, without a breakdown into C1 and C2. In the Turkish cohort, C0, C1, and C2 were present in 71.2% (290/408), 23.7% (97/408), and 5.1% (21/408) of patients, respectively, corresponding to crescents in 28.9% (118/408) of patients (C≥1). Combining these two cohorts, the crude pooled prevalence of any crescents (C≥1) was 27.6% (141/510). The Saudi cohort reported crescents only graphically, so it was not included in the pooled crescent estimate [7,9,10]. Overall, these findings indicate that adult IgAN in Middle Eastern cohorts is characterized by very frequent M1 lesions (~80%), high rates of S1 (~60%), more than half of patients harboring T1-T2 lesions (~56%), and crescents in approximately one quarter to one third of patients, where reported. The distribution of Oxford MEST-C components among included cohorts and crude pooled lesion frequencies is summarized in Table 4.

**Table 4:** Descriptive pooled frequencies of Oxford MEST-C lesions in the included Middle Eastern adult IgAN cohorts * Binomial 95% confidence intervals (Wilson) shown to indicate precision around the descriptive pooled proportion(sum of numerators/denominators), not a random-effects meta-analysis. † E1 counts were not extractable for the Saudi cohort [10]. ‡ Crescent counts were extractable only for the Turkish cohort [9]; Nasri 2012 [7] reported crescents only as an overall percentage without extractable counts/distribution, and [10] displayed crescents graphically without exact counts. Sensitivity (if Nasri’s “≈23% crescents” is approximated as 23/102): pooled C≥1 = 141/510 (27.6%), 95% CI 23.9-31.7.

Oxford lesion	Cohorts contributing (study IDs)	Patients contributing (N)	Pooled n/N (%)	95% CI*
M1	3 ((8–10))	528	422/528 (80.0)	76.3–83.1
E1	2 ((8–9))†	510	182/510 (35.7)	31.6–39.9
S1	3 ((8–10))	528	309/528 (58.5)	54.3–62.6
T1–2	3 ((8–10))	528	298/528 (56.4)	52.2–60.6
Any crescents (C≥1)	1 ((9))‡	408	118/408 (28.9)	24.7–33.5

Renal Outcomes

Reporting of renal outcomes was limited and heterogeneous across studies. The large Turkish cohort, which enrolled high-risk IgAN patients receiving immunosuppressive therapy, reported composite renal endpoints including end-stage kidney disease (ESKD) and a substantial decline in the eGFR over a median follow-up of approximately 2.5 years. In this cohort, more advanced tubular atrophy/interstitial fibrosis lesions (T1-T2) and the presence of crescents (C≥1) were associated with worse renal outcomes, consistent with international validation studies of the Oxford MEST-C classification. In contrast, the smaller Iranian and Saudi cohorts primarily focused on baseline clinicopathologic profiles, with minimal longitudinal follow-up data [7,8,10].

Because of differences in outcome definitions, follow-up durations, and adjustment for confounders, we did not perform a quantitative meta-analysis of effect estimates. Instead, we qualitatively observed that - as in non-Middle Eastern populations - tubular atrophy/interstitial fibrosis (T) lesions and crescents (C) appear to be the strongest histologic predictors of adverse kidney outcomes in Middle Eastern patients. Renal outcomes according to histologic lesions in the available cohorts are summarized in Table 4 [5,6,11,19-21].

Discussion

In this PRISMA-guided systematic literature review with crude pooled lesion frequencies from adult IgAN cohorts in Middle Eastern countries, we found that the histopathologic profile of IgAN in the region is dominated by mesangial hypercellularity, frequent segmental sclerosis, and a high burden of chronic tubulointerstitial damage and crescents. Crude pooled analyses from three cohorts (n = 528) showed that approximately 80% of patients had mesangial hypercellularity (M1) lesions, nearly 60% had segmental sclerosis (S1), and more than half had tubular atrophy/interstitial fibrosis grade 1-2 (T1-T2), while roughly one quarter to one third had crescents (C≥1). These findings are consistent with the central prognostic role of tubular atrophy/interstitial fibrosis (T) and crescents (C) lesions in global Oxford MEST-C-based prognostic studies.

These observations indicate that, at the time of kidney biopsy, many patients in the included Middle Eastern cohorts have substantial chronic histologic injury and, in a sizeable minority, crescents. Interpreting this pattern as delayed diagnosis or “late biopsy” is plausible but remains speculative, as these data cannot quantify diagnostic lead time and are derived from tertiary-care convenience cohorts. Alternative explanations include a more aggressive underlying phenotype in some populations (genetic/epigenetic susceptibility), environmental or contextual influences (e.g., infection burden and other exposures), and variation in clinical practice - particularly biopsy indications, referral pathways, and pathology reporting practices. Nevertheless, these findings underscore the need for earlier recognition of hematuria/proteinuria, timely nephrology referral, and prospective regional IgAN registries with standardized biopsy and outcome reporting [14,18].

Our results align qualitatively with international validation studies of the Oxford classification, which have consistently demonstrated that T lesions and crescents are among the strongest histologic predictors of kidney outcomes [18,19]. The high prevalence of T1-T2 and C lesions in Middle Eastern cohorts reinforces their prognostic importance in this regional context and supports the use of the Oxford MEST-C score for risk stratification. At the same time, the limited availability of adjusted outcome data and the predominance of retrospective single-center cohorts highlight the need for well-designed prospective registries and clinical trials in the region [22-25].

This review has several strengths. We focused on adult, biopsy-proven primary IgAN from Middle Eastern countries and required the use of the Oxford classification, thereby ensuring a degree of clinicopathologic comparability across studies [10,11,26-28]. We used duplicate study selection and data extraction and applied a standardized risk-of-bias assessment with the NOS. We also derived simple, crude pooled lesion frequencies to provide a pragmatic summary of MEST-C distributions, which may help clinicians and policymakers appreciate the spectrum of IgAN severity in the region [12,13,27-29].

However, several important limitations must be acknowledged. First, the number of eligible studies was small, and key Middle Eastern countries - including many Gulf, Levant, and North African nations - were not represented, limiting generalizability across the broader region. Second, all cohorts were retrospective and based on center-level convenience samples of biopsied patients, with a likely over-representation of more severe cases; hence, our crude pooled proportions cannot be interpreted as population-level prevalence estimates for Saudi Arabia, Iran, or Turkey. Third, reporting of clinical variables, MEST-C components, and renal outcomes was heterogeneous, and some data had to be approximated from reported percentages. Histopathologic assessment was performed locally in each study without central re-reading, and inter-observer agreement statistics (e.g., kappa coefficients) were not reported, precluding formal assessment of diagnostic heterogeneity. We could not consistently distinguish between C1 and C2 lesions or between T1 and T2 lesions in all cohorts and therefore focused on combined categories where necessary. Finally, the very small number and clinical diversity of cohorts meant that conventional meta-analytic statistics for heterogeneity and small-study effects could not be meaningfully applied; we therefore deliberately restricted our quantitative synthesis to crude pooled lesion frequencies and a narrative summary of renal outcomes.

Despite these limitations, this review provides, to our knowledge, the first structured overview of adult IgAN clinicopathology in Middle Eastern cohorts using the Oxford MEST-C classification. The findings suggest that advanced chronic damage and crescents are common at the time of biopsy in the available cohorts, reinforcing the prognostic importance of T and C lesions in this regional context and supporting the use of the MEST-C score for risk stratification. At the same time, the descriptive nature of our pooled lesion frequencies and the reliance on retrospective convenience samples underscore the need for earlier identification of urinary abnormalities, standardized biopsy and reporting practices, and well-designed prospective regional IgAN registries and clinical trials in the Middle East.

## Conclusions

Adult primary IgAN in the limited Middle Eastern cohorts identified in this review appears to be characterized by a high frequency of mesangial hypercellularity (M1) and segmental sclerosis (S1), with moderate-to-severe tubular atrophy/interstitial fibrosis (T1-T2) and crescents present in approximately one quarter to one third of biopsied patients. These observations are compatible with - but do not prove - delayed detection and referral in some centers. Although our data cannot quantify diagnostic lead time or provide population-level prevalence estimates, they underscore the importance of earlier identification of hematuria and proteinuria, timely nephrology referral, and the development of prospective regional IgAN registries and clinical trials to refine risk stratification and improve outcomes for patients with IgAN in the Middle East.

## Appendices

**Table 5:** Database search strategies

Database search strategies
Databases searched: MEDLINE (via PubMed), Embase, and Scopus.
Search window: January 1, 2009, to November 24, 2025 (date of last search).
Language restrictions: None.
Study type limits: None applied at search stage.
Population limits: Searches were limited to human studies where applicable; adult-only restriction was applied at screening (eligibility criteria: ≥18 years).
Geographic terms: Searches included individual Middle Eastern country names (Saudi Arabia, Kuwait, Qatar, United Arab Emirates/UAE, Oman, Bahrain, Jordan, Lebanon, Syria, Iraq, Yemen, Egypt, Iran, Turkey).
Supplementary searching: Backward reference screening of eligible studies and relevant reviews; forward citation tracking using Google Scholar and/or Web of Science.

**Table 6:** PubMed (MEDLINE via PubMed) search

PubMed (MEDLINE via PubMed)
( ("Glomerulonephritis, IgA"[Mesh] OR "IgA nephropathy"[tiab] OR "IgA Nephropathy"[tiab] OR "Berger disease"[tiab] OR "Berger's disease"[tiab] OR "IgA glomerulonephritis"[tiab]) AND (Oxford[tiab] OR MEST[tiab] OR "MEST-C"[tiab] OR "Oxford classification"[tiab]) AND ("Saudi Arabia"[tiab] OR Saudi[tiab] OR Kuwait[tiab] OR Qatar[tiab] OR "United Arab Emirates"[tiab] OR UAE[tiab] OR Oman[tiab] OR Bahrain[tiab] OR Jordan[tiab] OR Lebanon[tiab] OR Syria[tiab] OR Iraq[tiab] OR Yemen[tiab] OR Egypt[tiab] OR Iran[tiab] OR Turkey[tiab]) ) AND ("2009/01/01"[Date - Publication] : "2025/11/24"[Date - Publication]) AND Humans[Mesh]

**Table 7:** PubMed (MEDLINE via PubMed) search

PubMed (MEDLINE via PubMed)
Query (run November 24, 2025): ( ("Glomerulonephritis, IgA"[Mesh] OR "IgA nephropathy"[tiab] OR "IgA Nephropathy"[tiab] OR "Berger disease"[tiab] OR "Berger's disease"[tiab] OR "IgA glomerulonephritis"[tiab]) AND (Oxford[tiab] OR MEST[tiab] OR "MEST-C"[tiab] OR "Oxford classification"[tiab]) AND ("Saudi Arabia"[tiab] OR Saudi[tiab] OR Kuwait[tiab] OR Qatar[tiab] OR "United Arab Emirates"[tiab] OR UAE[tiab] OR Oman[tiab] OR Bahrain[tiab] OR Jordan[tiab] OR Lebanon[tiab] OR Syria[tiab] OR Iraq[tiab] OR Yemen[tiab] OR Egypt[tiab] OR Iran[tiab] OR Turkey[tiab]) ) AND ("2009/01/01"[Date - Publication]: "2025/11/24"[Date - Publication]) AND Humans[Mesh]

**Table 8:** Embase (Elsevier) search

Embase (Elsevier)
Query (run November 24, 2025): ('iga nephropathy'/exp OR 'iga nephropathy':ti,ab OR 'berger disease':ti,ab OR "berger's disease":ti,ab OR 'iga glomerulonephritis':ti,ab OR 'glomerulonephritis iga':ti,ab) AND (oxford:ti,ab OR mest:ti,ab OR 'mest-c':ti,ab OR 'oxford classification':ti,ab) AND (saudi:ti,ab OR "saudi arabia":ti,ab OR kuwait:ti,ab OR qatar:ti,ab OR "united arab emirates":ti,ab OR uae:ti,ab OR oman:ti,ab OR bahrain:ti,ab OR jordan:ti,ab OR lebanon:ti,ab OR syria:ti,ab OR iraq:ti,ab OR yemen:ti,ab OR egypt:ti,ab OR iran:ti,ab OR turkey:ti,ab) AND [humans]/lim AND 2009-2025]/py

**Table 9:** Citation search

Citation search
Reference lists of all eligible studies and relevant reviews were screened (backward citation searching). Forward citation tracking was performed for included studies using Google Scholar and/or Web of Science to identify additional or more recent reports.
